# Evaluation of diagnosed cases of eye rhinosporidiosis in a public hospital of Maranhão, Northeast Brazil

**DOI:** 10.1186/s12886-019-1223-x

**Published:** 2019-11-08

**Authors:** Francílio Araújo Almeida, Antonio Augusto Lima Teixeira-Junior, Jaqueline Diniz Pinho, Elaine Fiod Costa, Gyl Eanes Barros Silva

**Affiliations:** 1 Laboratory of Immunofluorescence and Electron Microcopy (LIME), Presidente Dutra University Hospital, Federal University of Maranhão, São Luís, Maranhão Brazil; 2 Postgraduate Program in Adult Health (PPGSAD), Federal University of Maranhão, São Luís, Maranhão Brazil; 3 Department of Medicine I, Federal University of Maranhão, São Luís, Brazil; 4 Department of Pathology, Ribeirão Preto Medical School, University of São Paulo, Ribeirão Preto, São Paulo, Brazil; 5 Barão Itapary Street, 227, 65020 070, São Luís, Maranhão Brazil

**Keywords:** Oculosporidiosis, Rhinosporidiosis, Maranhão

## Abstract

**Background:**

Oculosporidiosis (ocular rhinosporidiosis) accounts for 15% of cases of rhinosporidiosis, which is a chronic granulomatous disease and is endemic in India and Sri Lanka. In Brazil, the climatic and hydrographic similarities to these endemic areas and the presence of riverside populations contributes to an increase in the incidence of rhinosporidiosis particularly in the State of Maranhão. This study, therefore, aimed to identify the number of diagnosed cases of oculosporidiosis and describe its the clinical epidemiology, laboratory, histopathology, and therapeutic characteristics.

**Methods:**

The study is descriptive, observational, and cross-sectional, and reports the prevalence and clinical epidemiological characteristics of oculosporidiosis in the State of Maranhão, Brazil. A retrospective analysis of the paper and electronic records for a period from 1999 to 2017 was conducted in the University Hospital of Federal University of Maranhão (HU-UFMA), located in the northeastern region of Brazil.

**Results:**

Thirty patients were diagnosed with rhinosporidiosis, eight of them had oculosporidiosis and seven of these met the criteria to be included in the study. Of the cases (23.3% of all 30), five were men (71.4%) and two women (28.5%), with an average age of 16.4 ± 15.6 years. In terms of race, four patients (57.1%) declared themselves white and three (42.9%) as brown. The north of the state, the mesoregion, had the most diagnosed cases accounting for 57.1% of the total. Left eye was the most affected site, reported in six patients (85.7%), while the conjunctiva was affected in all patients. Rhinosporidiosis and papilloma were the predominant diagnostic hypotheses (28.5 and 28.5%, respectively), followed by chronic scleritis, granuloma, and chalazion (14.25, 14.25, and 14.25%, respectively). All these cases were treated with lesion excision, and only two patients (28.5%) progressed with recurrence.

**Conclusion:**

It was verified that there was a male predominance, with only one eye reported as an infected site, with no bilateral involvement. The younger age group (between 1 and 2 years of age) was more affected by oculosporidiosis, and histopathological examination was necessary for a conclusive diagnosis.

## Background

Rhinosporidiosis is a chronic granulomatous disease caused by *Rhinosporidium seeberi*, categorized as microorganism protist of the class Mesomycetozoea, through ribosomal DNA analysis [1–3]. The most common sites of infection are the nose and nasopharynx (70%), followed by the eyes (15%). The rare sites of involvement are the: penis, lips, skin, and uvula [4–6].

The disease has the highest number of cases in India and Sri Lanka [5]. In Brazil, the great majority of the cases are noted in the State of Maranhão [7–10]. Rhinosporidiosis mainly affects males and has a predominance between the second and fourth decades of life [6, 11, 12].

Transmission of the disease occurs through the traumatized epithelium (transepithelial infection), which comes in contact with contaminated water and / or soil [3, 13]. Patients subsequently develop a mass of polypoid traits, which is pedunculated, and associated with bleeding, pruritus, and sneezing [11, 14].

In the ocular region, the conjunctiva and lacrimal sac are the most affected areas, representing between 50 to 77.6% and 24 to 33% of cases, respectively. In addition, the sclera and eyelid are also affected by oculosporidiosis [1, 10, 15–17]. In both tarsal and bulbar sides may be affected in the conjujctiva, with predominance in the tarsal region [18].

Clinically, oculosporidiosis appears as a friable polypoid mass, causing a foreign body sensation, with irritation and watering from the eye, and with no effects on visual acuity according to some isolated case reports [3, 16, 19, 20].

A rare complication of the lesion in the conjunctival region is the formation of staphyloma, which occurs due to scleral thinning and herniation of the intraocular content. This can lead to rupture and loss of the intraocular content, along with its infection due to scleral tissue dissolution, caused by the overlap of *R. seeberi* [21, 22].

The differential diagnosis includes mainly papilloma and pyogenic granuloma, which are treated with surgical excision of the lesion and cauterization of its base [13, 23, 24]. In association with surgery, dapsone is used to prevent the maturation of sporangia and promote fibrosis [25, 26]. The definitive diagnosis is obtained by histopathological examination, with demonstration of a fibrous stroma with several thick-walled sporangia at different stages of maturation and containing numerous endospores [24, 27, 28].

Although Brazil contains areas with high incidence of the disease, few cases have been reported in the country, mainly in the ocular form. Therefore, it was proposed to retrospectively raise the cases of this form of presentation of the disease in a specialized reference center in the State of Maranhão and to describe the clinical, epidemiological, laboratory, histopathological and therapeutic conduct characteristics, as well as to serve as bibliographical reference for professionals who have little knowledge about oculosporidiosis and rhinosporidiosis in general.

## Methods

The study is descriptive, observational, and retrospective study in nature. The study was developed at the Pathology Anatomy Unit of the University Hospital of the Federal University of Maranhão (HU-UFMA).

The oculosporidiosis cases were studied using the following data: epidemiological (such as age, sex, color, and place of residence), clinical aspects related to the lesion (such as location, time of evolution, and symptoms), and the histopathological examination, which included the complete diagnosis and treatment. The data were collected from information contained in the paper and electronic medical records. Patients with ocular lesions, that confirmed rhinosporidiosis on histopathological examination between 1999 and 2017, were enrolled in this study.

The exclusion criteria were unavailability of the medical records or paraffin block. The data were evaluated and transferred on to a Microsoft Excel 2010 spreadsheet, where results were arranged in a tabulated format.

## Results

**Fig. 1:**
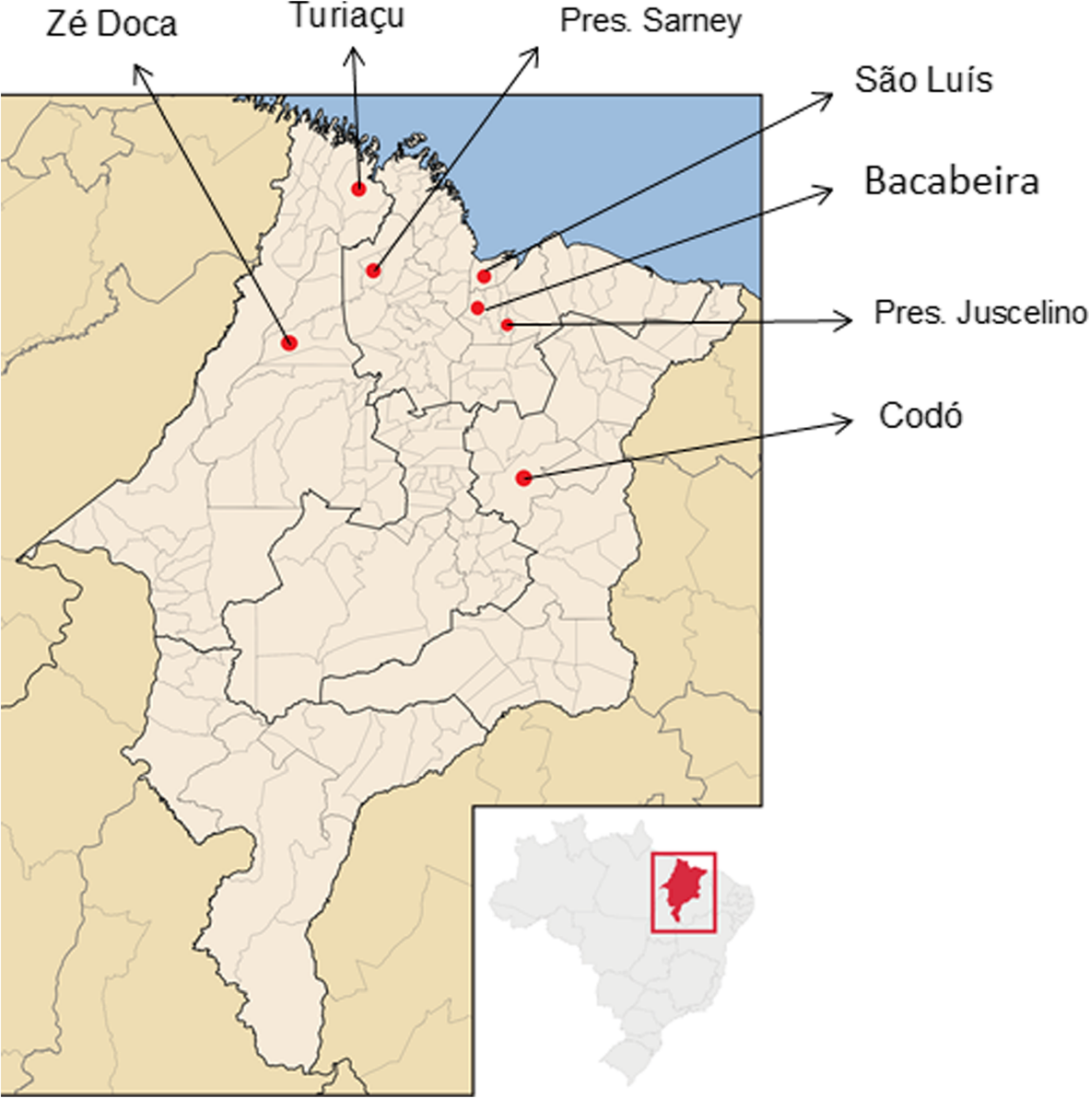
Geographic distribution of cases of oculosporidiosis between 1999 and 2017 in the State of Maranhão, Brazil (adapted from https://pt.wikipedia.org/wiki/Maranh%C3%A3o#/media/File:Maranhao_MesoMicroMunicip.svg)

Thirty patients with a diagnosis of rhinosporidiosis (through histopathological examination) were enrolled in this study, eight of these presented ocular involvement by the disease. One of the patients was excluded because of incomplete medical data. Thus, seven patients with oculosporidiosis were evaluated in this study, corresponding to 23.3% of the total number of patients with rhinosporidiosis. Of these, five were men (71.4%) and two women (28.5%), predominantly young, aged between 5 and 51 years: three in the first decade of life (42.9%), three in the second (42.9%), and one was in sixth decade of life (14.25%), with an average age of 16.4 ± 15.6 years. Regarding color or race, four declared themselves as white (57.1%) and three as brown (42.9%) (Table 1). As for the origin, the northern Maranhão, the mesoregion of the state, had a greater number of cases, four patients (57.1%) (Fig. 1).

**Table 1:** Clinical-epidemiological characteristics of patients with oculosporidiosis in HUUFMA, Maranhão (1999–2017)

Variables	N	%
Gender		
Male	5	71,4
Female	2	28,5
Age group		
1–10	3	42,9
11–20	3	42,9
51–60	1	14,25
Color		
White	4	57,1
Brown	3	42,9
Location		
São Luís	1	14,25
Other cities	6	85,7
Location of injury		
Left eye	6	85,7
Right eye	1	14,25
Diagnostic hypothesis		
Oculosporidiosis	2	28,5
Papilloma	2	28,5
Chronic Scleritis	1	14,25
Calazio	1	14,25
Granuloma	1	14,25

In six of the cases (85.7%), lesions were identified in the left eye, while only one (14.25%) had in the right eye. In all the cases, the conjunctiva was the primary lesion site, six were in the tarsal conjunctiva (85.7%) and one in the bulbar conjunctiva (14.25%). The disease progression described in the medical records (i.e. beginning of the injury until medical attention) was between 3 months and 3 years, with an average of 18.6 ± 13.8 months (not including episodes of recurrence).

**Fig. 2:**
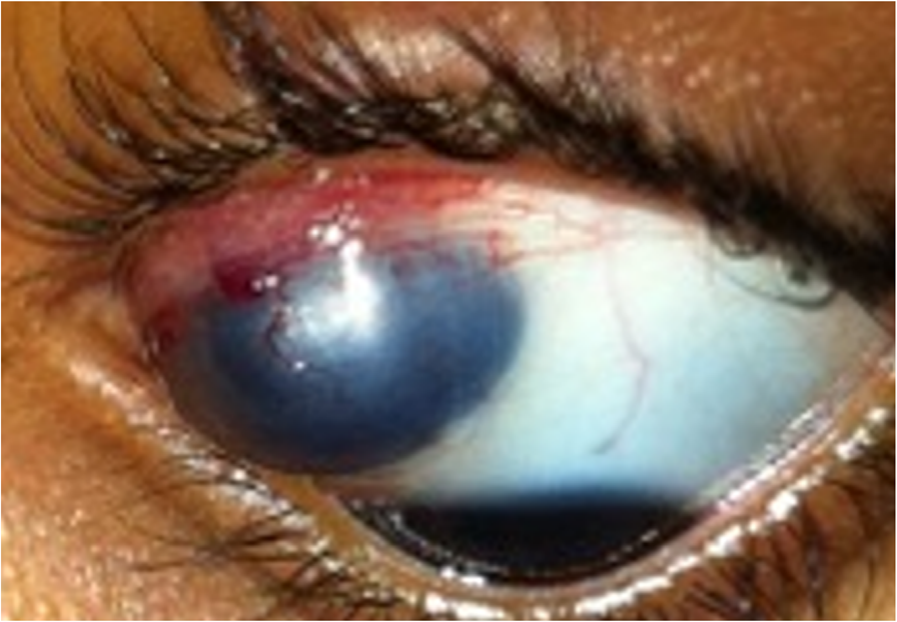
Conjunctival oculosporidiosis with staphoma in the left eye (image courtesy of Prof. Elaine de Paula Fiod Costa, UFMA)

The following symptoms were reported: episodes of bleeding, irritation, and decreased visual acuity (Table 2). The complication of staphyloma formation was observed in only one patient (14.25%) (Fig. 2). The diagnostic hypothesis was follows: rhinosporidiosis suspected in two patients (28.5%), papilloma in the other two (28.5%), while chronic scleritis, chalazion, and granuloma in one patient each (14.25%). There was no case of dacryocystitis and sinusitis reported. In the medical records, three patients (42.9%) had laboratory tests reported, with two presenting alterations in their eosinophil values: first one with 18% and 1702 mm^3^ (maximum reference values of 5% and 500 mm^3^), and the second with 5.9% and 655.49 mm^3^ (maximum reference values of 5% and 500 mm^3^).

**Table 2:** Clinical signs and symptoms of patients with oculosporidiosis in HUUFMA, Maranhão (1999–2017)

Variables	N	%
Bleeding	02	28,6
Rashes	02	28,6
Loss of sight	02	28,6
Not available	01	14,2

All patients were treated by surgical excision of the lesion, without the use of any complementary medication. Two patients (28.5%) had regular clinical follow-up: one underwent surgery 1 year and 3 month after the first one for staphyloma repair, while the other patient presented with two recurrences within the 1 year and 5 month period, with excisions being performed.

**Fig. 3:**
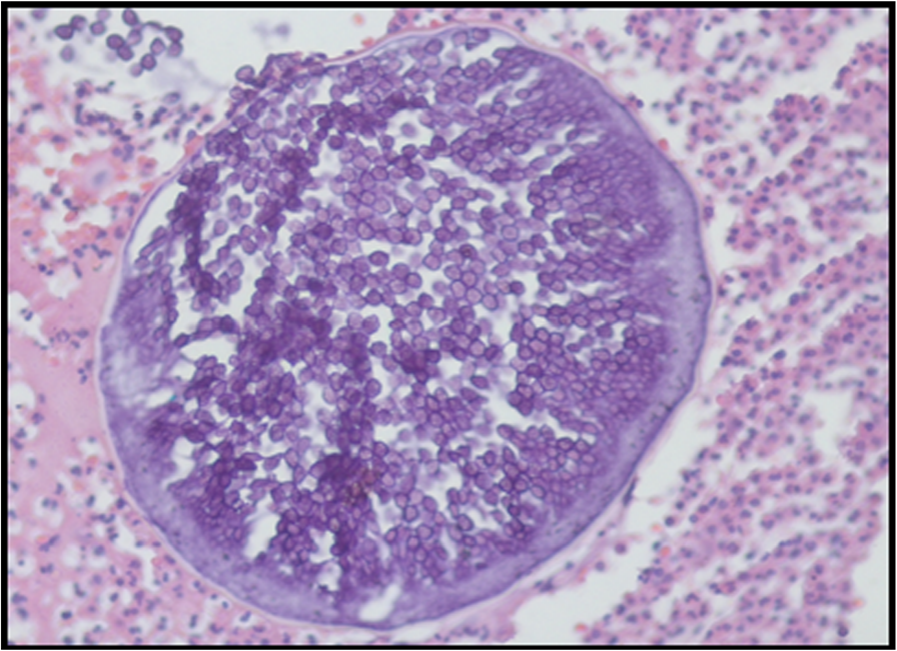
Sporangium containing several endospores surrounded by inflammatory infiltrate

The macroscopic aspects of all biopsies in the histopathological reports were described mainly as rough, brown, friable, and elastic consistency. The microscopic analysis of three patients reported, respectively: stratified squamous epithelial lining exhibiting mononuclear inflammatory infiltrate, with structures suggestive of *R. seeberi* sporangia (Fig. 3); pseudoepitheliomatous hyperplasia; and reactional hyperplasia of the squamous and glandular epithelium.

## Discussion

This study, involving a small sample, brings conformities with scientific articles, in both epidemiological and clinical factors. The ocular region represents on average 15% of cases of rhinosporidiosis, this study, however, presented a higher percentage than the literature data [22, 29]. The presumed mode of transmission of the disease from the natural aquatic habitat of *R. seeberi*, is through traumatized epithelium. The risk factors are related to the tropical climate zones, activities involving contact with soil or mud in a rural setting and bathing in still water [15, 17]. The higher frequency of cases among males could be related to rural activities, while among younger age groups could be ascribed to the habit of bathing in pond water [6, 28].

**Fig. 4:**
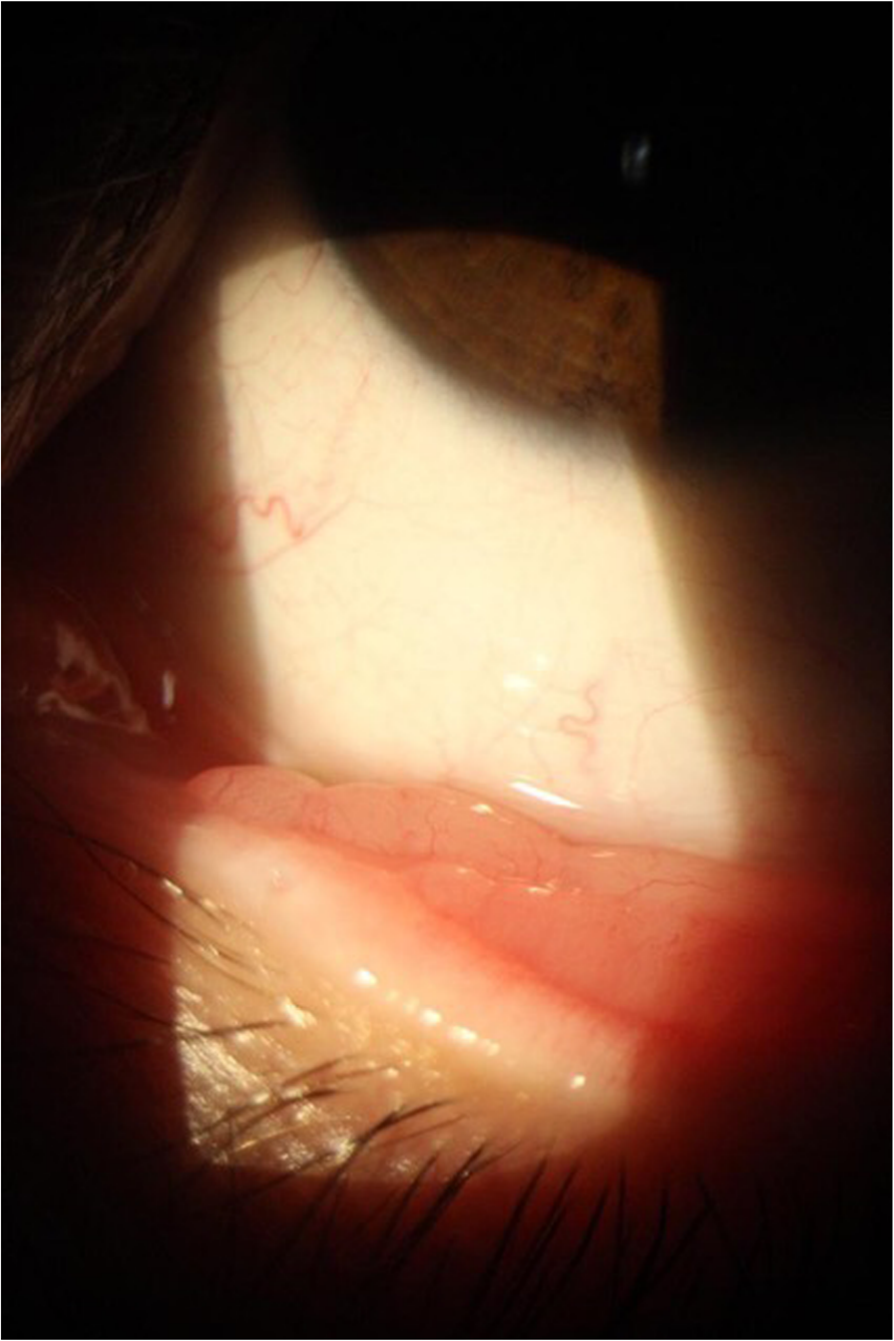
Oculosporidiosis of tarsal conjunctiva (image courtesy of Prof. Elaine de Paula Fiod Costa, UFMA)

The typical oculosporidiosis lesion is a freely mobile, granular pink or red, sessile or pedunculated lesion [17, 18]. There was only one-sided involvement. The conjunctiva is the site with the highest infection rate, with granuloma formation due to foreign matter deposited in the conjunctiva. In all of our cases, patients presented conjunctival lesions, mainly in the tarsal conjunctiva (see Fig. 4). The time of lesion evolution varies between 3 months and 1 year [16, 19, 22, 24, 30] with an average of 9 ± 5.09 months, a time relatively close to that found in the sample, which had an average evolution of symptoms around 18.6 months.

Visual impairment or loss in oculosporidiosis is a rarely seen as a clinical complaint or measured during the ophthalmologic examination. From the literature, 1 case of 5 that evaluates visual acuity, presented visual loss measured at the ophthalmological examination [1, 3, 16, 20, 21, 29]. While in our study, two of the patients reported visual complaint associated with ocular discomfort. The frequency of staphyloma, one of the complications of oculosporidiosis, is minimal, even in the endemic regions. It is also a recent finding, with the first cases recorded over 10 years, and according to analysis, there is an extension of the lesion to the retina [20–22]. One of the patients (14.25%) in our study developed this complication, requiring two surgeries for its correction.

The rates of suspecting rhinosporidiosis is low, considering it is a rare disease [2]. Suspicion is higher in countries where it becomes endemic, and thus physicians are more familiar with the disease [12, 15]. Regarding oculosporidiosis, this was considered a diagnostic hypothesis in 28.5% of our cases, a percentage similar to the papilloma, one of the main differential diagnosis.

Laboratory findings were not cited in most papers. In a case report of disseminated rhinosporidiosis, the patient presented 40% eosinophilia [4]. In another study, 10 patients out of a total of 63 had eosinophilia; however, values were not cited in the paper [31]. Two out of three patients with laboratory tests in our study revealed eosinophilia, and one of these showed a high increase with the maximum reference value. In such cases, the diseases with the same laboratory findings, such as parasitosis, however, cannot be ruled out [4].

Surgical removal of the lesion is the treatment of choice for oculosporidiosis, and it is recommended to use the adjunctive dapsone therapy to prevent sporangia maturation and promote stroma fibrosis [6, 13, 17]. Ocular recurrences are typically rare, with 1% rate for the conjunctiva, and between 2 and 40% for the lacrimal sac [26, 27, 32]. The causes: are incomplete excision of the lesion and spreading of endospores to the adjacent sites during a surgical procedure [14, 33]. The ineffective cauterization of the base of the lesion and failure to use dapsone in the postoperative period may make the lesion more susceptible to relapses. Despite being a benign condition, oculosporidiosis should be treated, as some cases can be lingering, locally invasive and devastating [16, 26, 32].

In the histopathological examination, *R. seeberi* is observed at all stages of development, and it is not possible to correlate some specific form of evolutionary stage with clinical presentation [34]. The literature describes some histological findings associated with infection, citing squamous metaplasia and epithelial hyperplasia [9, 19, 25].

## Conclusion

In our study, we described a large oculosporidiosis rate, one of the higher available in the literature (23.3%). Even though oculosporidiosis is not frequently diagnosed, it should be included as a possible underlying cause of chronic tarsal conjunctival lesions in countries with climate and rural activities. Habit to bath in pounds and stagnant water were associated with the infection. The clinical diagnosis of rhinosporidiosis is not easy (mainly in the ocular form), the histopathological examination is essential to confirm the diagnosis. The eosinophilia observed in the blood count may be a factor that helps in the diagnosis. Treatment of the disease is simple, and its prognosis is good.The research developed aims to provide more clinical and epidemiological information, complementing the scarce existing theoretical reference, so that health professionals may have more knowledge about this disease existing in the region, and its forms of presentation.

## Data Availability

The datasets used and/or analyzed during the current study available from the corresponding author on reasonable request.

## Abbreviations

HU-UFMA: University Hospital of Federal University of Maranhão
*R. seeberi*: *Rhinosporidium Seeberi*
